# Distinct functions of dynamin isoforms in tumorigenesis and their potential as therapeutic targets in cancer

**DOI:** 10.18632/oncotarget.16678

**Published:** 2017-03-29

**Authors:** Jianghui Meng

**Affiliations:** ^1^ Charles Institute of Dermatology, School of Medicine and Medical Sciences, University College Dublin, Belfield, Dublin, Ireland; ^2^ International Centre for Neurotherapeutics, Dublin City University, Glasnevin, Dublin, Ireland

**Keywords:** endocytosis, tumor, cancer target, clathrin, amphiphysin

## Abstract

Dynamins and their related proteins participate in the regulation of neurotransmission, antigen presentation, receptor internalization, growth factor signalling, nutrient uptake, and pathogen infection. Recently, emerging findings have shown dynamin proteins can also contribute to the genesis of cancer. This up-to-date review herein focuses on the functionality of dynamin in cancer development. Dynamin 1 and 2 both enhance cancer cell proliferation, tumor invasion and metastasis, whereas dynamin 3 has tumor suppression role. Antisense RNAs encoded on the DNA strand opposite a dynamin gene regulate the function of dynamin, and manipulate oncogenes and tumor suppressor genes. Certain dynamin-related proteins are also upregulated in distinct cancer conditions, resulting in apoptotic resistance, cell migration and poor prognosis. Altogether, dynamins are potential biomarkers as well as representing promising novel therapeutic targets for cancer treatment. This study also summarizes the current available dynamin-targeted therapeutics and suggests the potential strategy based on signalling pathways involved, providing important information to aid the future development of novel cancer therapeutics by targeting these dynamin family members.

## INTRODUCTION

**Figure 1:**
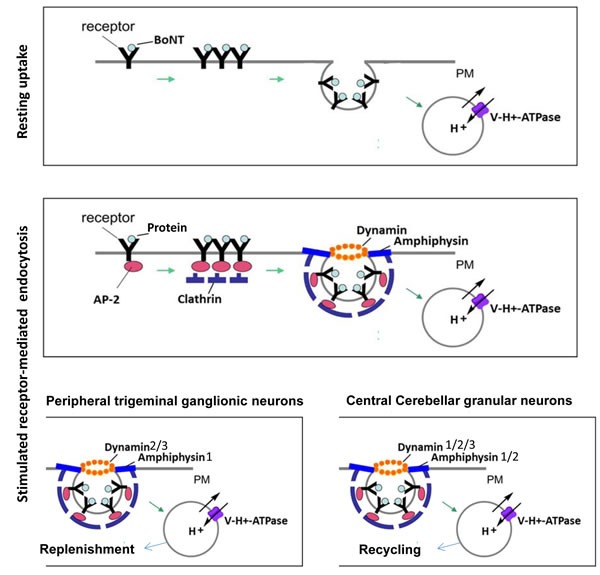
Schematic diagram for resting and stimulated receptor-mediated endocytosis in neurons and differential involvement of dynamin isoforms in stimulated receptor-mediated endocytosis Using a molecular probe, such as botulinum neurotoxin (BoNT) which cleaves SNARE proteins, to dissect the path of receptor-mediated endocytosis [115], it was revealed that resting uptake by neurons occurs via lipid rafts, acidified compartments and protein acceptors but not dynamin. In contrast, stimulated endocytosis of BoNT by neurons utilises lipid rafts, acidified compartments (Vacuolar-type H+ -ATPase) and dynamin and amphiphysin [4]. In terms of fast recycling of small clear synaptic vesicles, cerebellar granule neurons (CGNs) use predominantly dynamin 1, whereas isoform 2 and, to a lesser extent, isoform 3 to support a less rapid mode of stimulated endocytosis. In contrast, large dense-cored vesicle (LDCV)-releasing trigeminal ganglionic neurons (TGNs) preferentially employ dynamins 2 and 3 and amphiphysin 1 for evoked endocytosis. PM = plasma membrane, V-H+ = Vacuolar-type H+, CCP = clathrin coated pit, CME = clathrin mediated endocytosis.

Cancer is associated with features of dysregulated cell proliferation and increased migration of tumor cells, resulting in aggressive cell invasion and metastatic disorders. Dysregulation of endocytosis in cancer cells alters cell surface expression of critical molecules, leading to the dysfunction of cell signaling, migration, survival and proliferation. Endocytosis is critical for maintaining neurotransmission, antigen presentation, receptor internalization, growth factors signalling, nutrient uptake, and pathogen infection. In many cell types, multiple endocytotic pathways exist which differ in the proteins utilised and molecules endocytosed [1]. Dynamins support endocytosis by either clathrin-dependent or -independent mechanisms [1, 2]. In the case of receptor-mediated endocytosis, dynamins, clathrin, clathrin adaptor proteins (i.e. adaptor protein complex) as well as amphiphysins are essential. During endocytosis, receptor binding is mediated by clathrin adaptors that can bind directly to both clathrin and the lipid and/or protein components of membranes. Clathrin forms a triskelion shape composed of three clathrin heavy chains and three light chains, which is the assembly unit of a clathrin coated pit [3]. Not all the endocytosis requires clathrin, for example, in quiescent nerve endings a slow endocytosis can occur independent of dynamin and clathrin, however, in an intense stimulation condition, a fast endocytosis can occur as stimulation recruits dynamin, clathrin, clathrin adaptor protein 2 (AP2) complex, and amphiphysin to increase protein internalization (Figure 1). Moreover, numerous studies have shown that dynamin-independent and -dependent membrane recycling are independent processes linked to spontaneous and evoked exocytosis, respectively [4].

## THERAPEUTIC POTENTIAL OF DYNAMIN ISOFORMS

**Figure 2:**
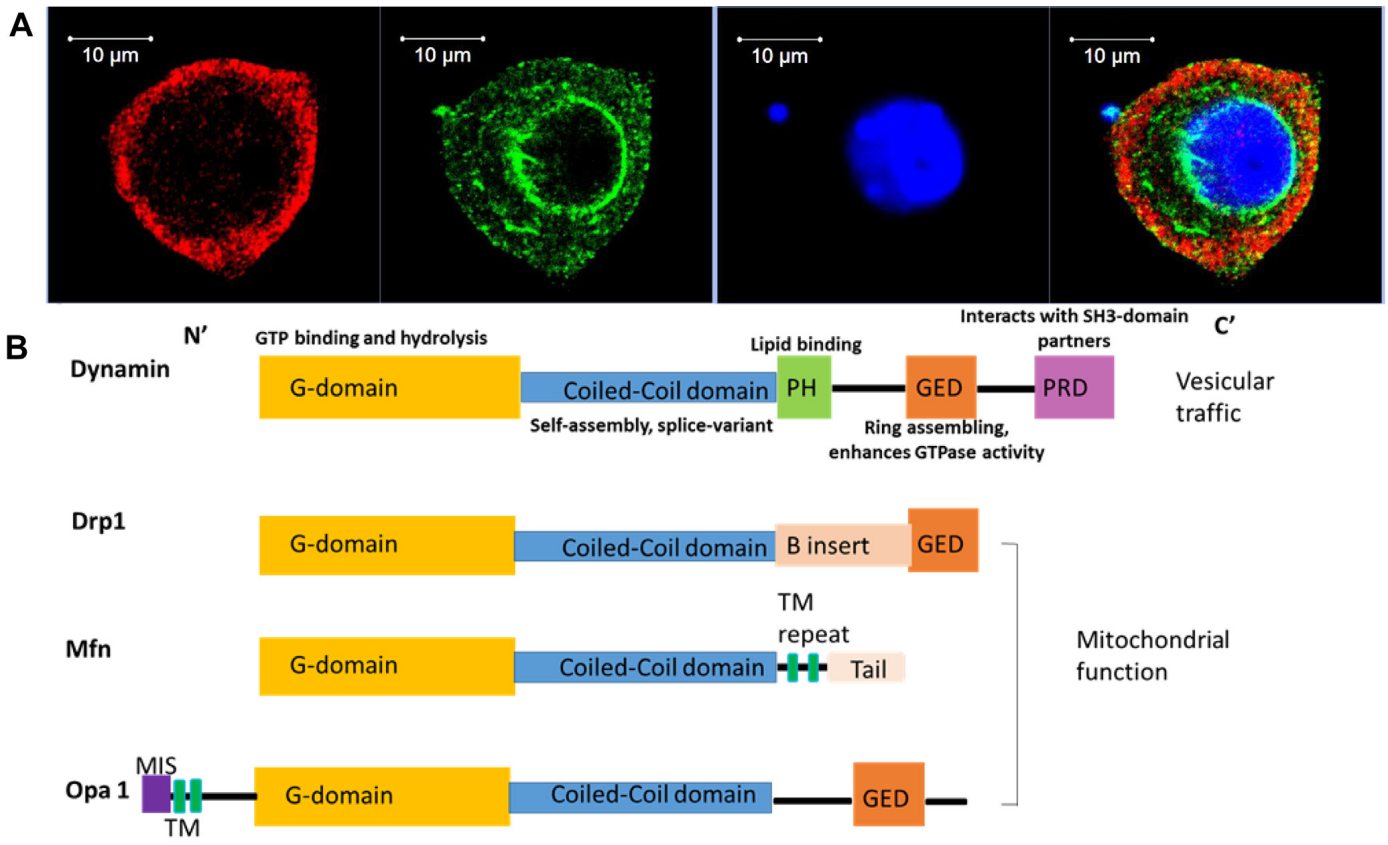
Distinct localization of dynamin isoforms in the peripheral neurons highlights their distinct functional importance The linear domain arrangement of human dynamin and related proteins. **A**. Immuno-fluorescence study demonstrated that dynamin 1 and 2 showed distinct distribution pattern in the cultured trigeminal ganglionic neuron. Dynamin 1, green; dynamin 2, red; nuclei, blue. **B**. Each domain of dynamin protein is indicated as follows: G domain, yellow, is responsible for GTP binding and hydrolysis; middle coiled-coil domain– GTPase Effector Domain (GED) stalk, blue, is responsible for dynamin protein self-assembly, and variant splicing; PH domain (pleckstrin homology), green, is responsible for lipid binding; GED, orange, is responsible for ring assembling and also enhance GTPase activity; PRD (proline rich domain), light purple, interacts with SH3-domain partners, such as amphyphisin; N-terminal MIS (mitochondrial import sequence): dark purple, is responsible for targeting the OPA1 protein; B insert domain (pink) of the Dnm1 guanosine triphosphatase (a Drp) contains a novel motif required for association with the mitochondrial adaptor Mdv1 to the mitochondria.

Dynamins are large GTPase, encoded by three genes in mammals, required for vesicle recycling and membrane fission. Dynamins can be directed by amphiphysins to sites of endocytosis [5, 6], where they form a helix around the neck of the invaginated coated pit. They can self-assemble to form ring or helix-like structures around the neck of the vesicle and play an essential role in scission of the invaginated vesicles during membrane fission (Figure 1). Three conventional mammalian dynamin genes (DNM1, DNM2 and DNM3) encode proteins that are ~80% homologous [4]. Dynamin 1 is generally considered to be neuron specific, but a recent finding showed it can be activated to mediate rapid compensatory clathrin-mediated endocytosis (CME) in non-neuronal cells [7, 8]. In contrast, dynamin 2 is ubiquitously expressed and 3 is mainly expressed in the testis, brain and low levels in the lung [9]. Despite sharing significant sequence identity, dynamin 1 and 2 differ in their curvature-generating/sensing properties [10]. In the peripheral sensory neurons, dynamin 1 and 2 have been found to have distinct localization, with isoform 1 located in the peri-nuclear region and 2 mainly resides on the plasma-lemma, suggestive of the distinct functions for these two isoforms (Figure 2A). Dynamin 1 has more potent curvature-generating properties, and it is better suited for rapid, compensatory endocytosis at the synapse, whereas the curvature-sensing properties of dynamin 2 require a narrow neck to trigger its assembly, therefore, dynamin 2 is more suited to monitor and regulate maturation of the early stages of clathrin-coated pits (CCPs) [10]. There has been reported evidence for isoform-specific functions of dynamin 1 and 2 as well as 3 in clathrin-mediated endocytosis both at the central synapse [11], peripheral nerves [4] and non-neuronal cells [12], respectively (Figure 1). Moreover, an overlapping but distinct role between 3 isoforms of dynamin has been shown, for example, expressing any of the three dynamin isoforms in dynamin 1 knock-out (KO) neurons could compensate for the dynamin 1 phenotype [13].

Although the basis for isoform differences and their functional significance remains poorly understood, each isoform has been linked to different disease conditions, including Alzheimer's disease, Parkinson's disease, Huntington's disease, Charcot-Marie-Tooth disease, heart failure, schizophrenic, epilepsy dominant optic atrophy, osteoporosis, Down's syndrome and various types of cancer [14] (Table 1).

**Table 1:** Influence of dynamin and related proteins on cancergenesis

Dynamin isoforms	Cancer cell types with abnormally expressed or activated dynamin proteins	Outcome	
Dynamin 1	Acute myeloid leukemia, lung and colon adenocarcinomas, H1299 non-small lung cancer cells	Aberrant trafficking of nascent clathrin-coated vesicles and alteration of cell signalling and enhancement of cell proliferation; dynamin 1 is acutely activated by an Akt/GSK3β signalling cascade to increase the rate of CCP initiation in the H1299 cancer cells.	[7, 8, 30]
Dynamin 2	Progressive prostate cancer (PCA), pancreatic tumor cells, hepatocellular carcinoma (HCC), colon-derived tumor cell line, HCT116, pancreatic tumor cells, malignant glioblastomas, breast-cancer cells, human non-small cell lung carcinoma cells, invasive bladder cancer cells, adult T-cell acute lymphoblastic leukemia	Dynamin 2 expression is significantly increased in these cancer conditions. Dynamin 2 is involved in the promotion of cytokinesis, enhancement of tumor invasion and metastasis. Dynamin 2 overexpression is associated with poor prognosis; Dynamin 2 and cortactin participate in cell migration by stabilizing F-actin bundles in filopodia. Dynamin 2 contributes to the bladder cancer invasion by controlling invadopodia formation; Mutation of dynamin 2 in oncogenesis of T-cell acute lymphoblastic leukemia	[33, 37, 39, 42, 45, 99–103]
Dynamin 3	Hepatocellular carcinoma pathogenesisis	Dynamin 3 gene (DNM3) is hyper-methylated and protein expression level is decreased. Dynamin 3 has tumor suppressive function in HCC by upregulating and activating p53.	[46, 47]
Dynamin-related protein 1	Lung and breast cancers, glioblastoma cells, Human lung cancer cell; T-cell acute lymphoblastic leukemia cells	Drp1 is upregulated in certain types of cancers. Upregulated Drp1 confers chemotherapy resistance, induces apoptotic resistance and autophagy, facilities cell cycle progression, generates genome instability, promotes cell migration and induces poor prognosis.	[26, 67, 69, 70]
Mitofusin 1 and Mitofusin 2	Human lung cancer cells express imbalance of Drp 1/Mfn 2 expression (an increase in Drp-1 and decrease in Mfn-2 ); downregulation of Mfn2 in hepatocellular carcinoma cells	Mfn-1 and -2 induce mitochondrial fission. Overexpression of Mfn-2, Drp-1 inhibition, or Drp-1 knockdown results in a marked reduction of cancer cell proliferation and an increase in spontaneous apoptosis.	[23, 25–27, 104]
Dynamin-like GTPase optic atrophy 1	Lung adenocarcinoma cells and hepatocellular carcinoma	Opa1 is upregulated in various forms of cancer. cell cycle progression, genome instability, cell migration, poor prognosis	[25, 27, 75]

Apart from the above isoforms, dynamin-related family members also contribute to carcinogenesis or tumor suppression. These dynamin-related proteins include fission proteins, for example, the large self-assembling GTPase dynamins, related protein 1 (Drp1, also called DLP1) [15], mitochondrial fission 1 (Fis1) [16], fusion proteins (Mfn1, Mfn2) [17–20], and Optic Atrophy 1 (OPA1) [21–24]. These proteins are essential to maintain mitochondrial fission and fusion balance to provide necessary adenosine triphosphate (ATP) to neurons. It is notable that certain cancer patients revealed an imbalanced expression of dynamin-related proteins in fusion and fission which resulted in increased mitochondrial fragmentation. In particular, reduced protein expression levels of Mfn1, Mfn2, and Opa1 and high levels of Fis1 and DLP1/ Drp1 were observed in certain cancer conditions [23, 25–28] (Table 1).

### Dynamin 1 signalling in carcinogenesis

Dynamin 1 has been linked to many neurological diseases for example amyotrophic lateral sclerosis and Alzheimer's disease [29]. Expression of dynamin 1 is upregulated in a number of cancer cells from acute myeloid leukaemia, lung and colon adenocarcinomas [30], perhaps reflecting a protective function against pathways of apoptosis (Table 1). It has been shown most recently that overexpression and activation of dynamin-1 is selectively activated down-stream of tumor necrosis factor (TNF)-related apoptosis-inducing ligand (TRAIL)–death receptors to self-regulate their endocytosis, attenuate apoptotic signalling, enhance cells survival and migration [8]. In another word, a reduced level of dynamin-1 expression confers greater sensitivity to TRAIL-induced apoptosis, whereas enhanced level of dynamin-1 expression results more resistance to apoptosis. Dynamin 1 is also acutely activated by Akt/GSK3β signalling in H1299 non-small lung cancer cells, which results in an increase of the rate of CCP initiation, and alteration of CCP dynamics, as well as triggering rapid, accelerated calthrin-mediated endocytosis (CME) [7]. The dysregulated CME leads to aberrant trafficking of nascent clathrin-coated vesicles (CCVs), and alters cell signalling and enhances cell proliferation. This function is also the first evidence of dynamin 1 function in non-neuronal cells [7]. Altogether, dynamin 1- targeted anti-cancer therapeutics may potentially to induce cancer cell apoptosis.

### Dynamin 2 as a prognostic marker in cancer

Unlike dynamin 1, dynamin 2 has been found to be expressed ubiquitously. It is best known for its role in membrane trafficking processes and, recently, it has been uncovered to promote the cytokinesis, cell proliferation, migration, and act as prognostic marker. Dynamin 2 is required for the abscission phase of cytokinesis. Knockdown of dynamin results in multinucleation, a characteristic of a failed cytokinesis in Hela cells [31]. In terms of endocytosis, it is well known that the endocytosis of epidermal growth factor receptor (EGFR) requires dynamin 2. EGFR induces tumorigenesis by multiple signalling pathways. Amplification of both dynamin 2 and EGFR genes have been demonstrated to occur in carcinoma patients [32]. After dynamin 2 is knocked down or inhibited, the internalization of EGFR is delayed and reduced, resulting in increased EGF-mediated tumor cell migration, colony formation and invasion [33, 34]. Moreover, dynamin 2 is necessary for the endocytosis of several proteins associated with cancer motility and invasiveness, including integrin β-1 and focal adhesion (FA) kinase [35, 36]. Knockdown dynamin 2 leads to impaired focal adhesion disassembly and cell migration. Dynamin 2 also regulates polyamine internalization in a colon-derived tumor cell line, HCT116 [37]. Polyamines are ubiquitous small basic molecules involved in primordial stress resistance and their uptake is increased in cancer cells [38]. Polyamine uptake process is independent of clathrin but it is dependent on caveolin-1, highlighting dynamin 2 regulates clathrin-independent endocytosis in cancer cells.

Dynamin 2 expression was found to be upregulated in several cancerous conditions. Its overexpression is closely associated with neoplastic prostate epithelium and its poor prognosis [39]. In isolated progressive prostate cancer (PCA) cells, dynamin 2 was found to regulate focal adhesion turnover, which is critical for cell migration [39]. Elevated dynamin 2 expression levels are associated with Gleason score, tumor volume, and PCA-specific mortality. Knockdown of dynamin 2 expression or inhibition of dynamin 2 prevents cell invasiveness in androgen-responsive and -refractory PCA models, supporting the potential benefit of dynamin 2 to serve as a target for development of novel therapeutics for treatment of advanced PCA [39]. In preclinical models, dynamin 2 gene silencing significantly reduced cell migration and invasion *in vitro*, as well as tumor size and lymph node metastases *in vivo* [39]. Level of dynamin 2 was also found to be increased in the cervical cancer, which might be related to the increased proliferation, dysfunction of apoptotic activity and increased migration [40]. Together these results suggest that both enzymatic activity and proper localization of dynamin 2 are required for extracellular matrix degradation by invasive cancer cells [41].

**Figure 3:**
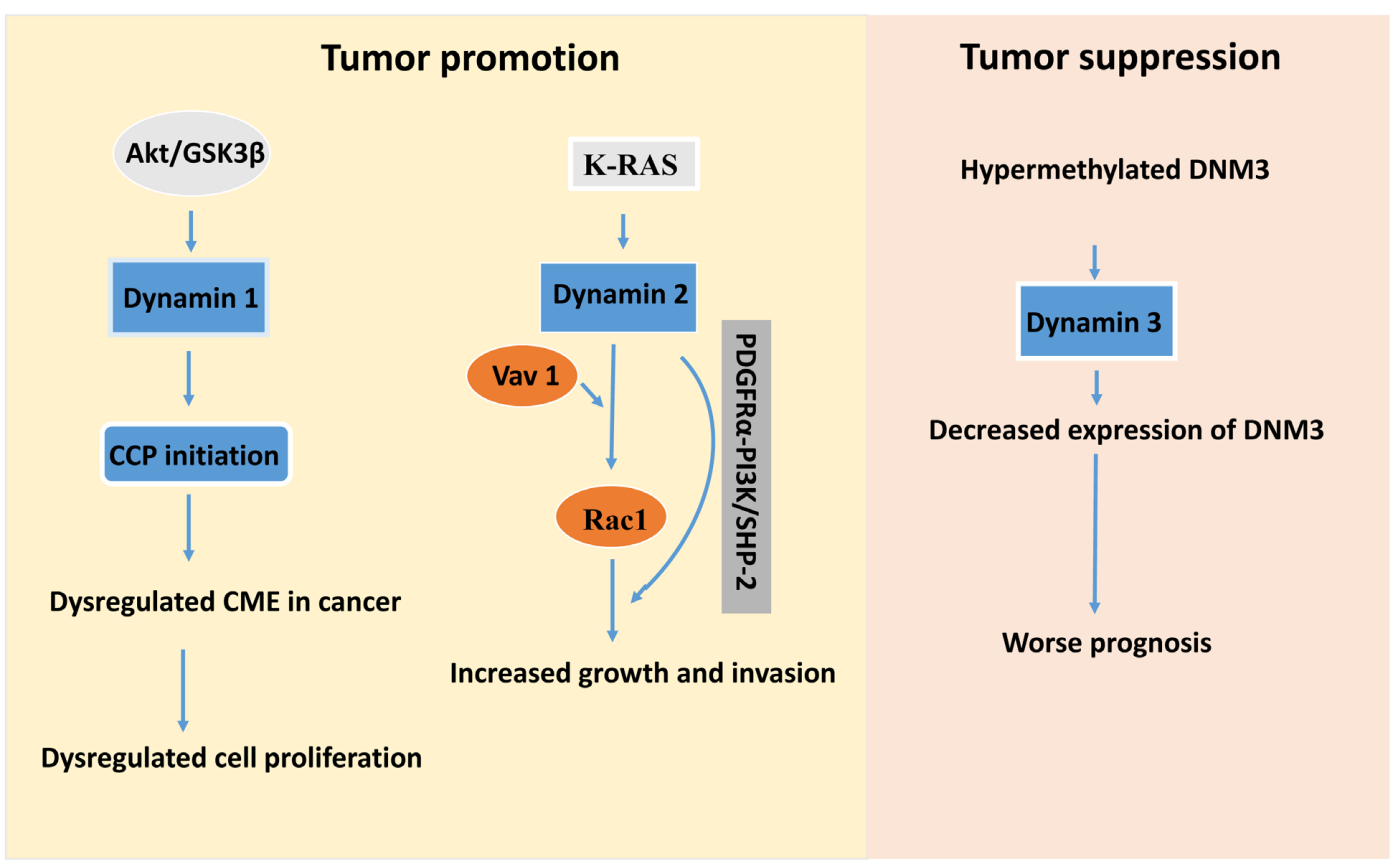
Schematic map for potential targets in dynamin-mediated tumor development whereas dynamin 1 and 2 act as the tumor promotors In contrast, dynamin 3 has tumor suppressive role. Inhibition or depletion of the Akt/GSK3β signalling pathway will prevent the function of dynamin 1 in lung cancer cells. Moreover, inhibition of dynamin 2 interaction with Vav1 will stop the activation of Rac1, and a method that prevents phosphorylation of PDGFRα-PI3K/SHP-2 will reduce the increased tumor growth and cancer cell invasion in certain types of cancer. Blockage of hypermethylation of DNM3 by some genetic approach or increase of expression of dynamin 3 protein will prevent the tumor development.

Different cell factors are demonstrated to be involved in the regulation of dynamin 2 function, for example, dynamin 2 is regulated by proto-oncogenic expression of K-RAS in human colon cancer cells [37] and promotes pancreatic cancer cell migration through activation of Rac1 [42]. Dynamin 2 has been identified as a Vav1-SH3 interacting protein which remodels actin in T cells [43] and this interaction is deemed specific to Vac1, rather than Vac2 or 3 (Figure 3). Vac1 also interacts with an actin regulatory scaffold protein Zyxin which is known to be involved in cell adhesion, cell migration and integrin function in T cells and control the cancer cell motility through integrin [44]. More recently, in pancreatic tumor cells, dynamin 2 was found to promote lamellipodia formation and pancreatic tumor cell migration by its direct binding with Vav1 to promote Rac1 activation and migration [42].

In brain cancer, dynamin 2 is a downstream effector of the PDGFRα-PI3K/SHP-2 signalling in glioma cells and mediates PDGFRα -SHP-2-promoted glioblastoma growth and invasion [45]. Depletion of endogenous dynamin 2 by short hairpin RNAs (shRNAs) inhibited PDGFRα-stimulated phosphorylation and activation of Akt, extracellular signal-regulated kinase 1/2, Rac1 and Cdc42, prevented glioma cell migration and tumor growth as well as invasion in the brains of mice. Thus, targeting dynamin 2 or PDGFRα-SHP-2-Dynamin 2 pathway may be beneficial to patients with malignant glioblastomas [45].

### Dynamin 3 as a tumor suppressor

Differing from dynamin 1 and 2, dynamin 3 can function as a suppressor of tumorigenesis by induction of p53 expression and activation [46]. Interestingly, dynamin 3 gene (DNM3) was identified as one of the genes associated with hepatocellular carcinoma pathogenesisis, from a triple combination array carried out in surgical specimens obtained from hepatocellular carcinoma (HCC) patients. 48 HCC patients were evaluated in that study for DNM3 methylation and expression status using methylation specific polymerase chain reaction (MSP-PCR) and semi-quantitative reverse transcriptase RT-PCR, respectively [47]. DNM3 was found being hypermethylated in cancer tissue compared with adjacent normal tissue, and this was associated with decreased expression of DNM3 in cancerous tissue (Figure 3). Patients with reduced expression of dynamin 3 in tumor tissues exhibited worse prognosis with decreased disease specific survival compared to patients without decreased expression [47].

## DYNAMIN GENE-RELATED NON-CODING RNAs ARE BIFUNCTIONAL

Non-coding RNAs are found to have a key function in the regulation of gene expression at the post-transcriptional level by base pairing with complementary regions mainly within the 3’ untranslated regions (3’-UTR) of target mRNAs, thus promoting mRNA degradation and translational repression. These intronic MicroRNAs (MirRNAs) can support the function of its host gene by silencing genes that are functionally antagonistic to the host, or act synergistically with the host by coordinating the expression of genes with related functions. MirRNAs promote or inhibit cancer growth by regulate the expression of target genes. In relation to the dynamin genes, MicroRNA-214 (MiR-214) is located about 6kb apart on chromosome lg24.2 in an intron of the DNM3 gene, and is also involved in carcinogenesis. Although the biological function of MiR-214 still remains unclear in certain cancers, MiR-214's pleiotropic and tumor-specific contribution to various cancer hallmarks formation and progression is achieved via several target genes [48]. It functions as both a tumor suppressor and oncogene in various types of human cancers. Moreover, MiR-214 is a novel biomarker for lymph node metastasis in patients with gastric cancer and plays an important role in the development of gastric cancer in the Chinese Han population [49]. In samples from breast cancer that resistant to cisplatin and tongue squamous carcinoma cell lines as well as in ovarian cancer resistant to platinum, MiR-214 was found to be over-expressed [50–52]. But, in the cervical cancer tissues [53], multiple melanoma [54], ovarian [55], hepatocellular carcinomas [56] and bladder cancer [57], miR-214 is frequently down-regulated. A high MiR-214 level was also detected in the non-small cell lung cancer cell lines resistant to doxorubicin [58]. In general, overexpression of MiR-214 inhibited cancer cell proliferation and induced apoptosis [55]. MiR-214 itself is also transcriptionally regulated by transcription factors, peroxisome proliferator-activated receptor-α (PPARα), hypoxia-inducible factor-1α (HIF-1α), and it also is involved in the post-transcriptional regulation of gene expression in multicellular organisms by affecting both the stability and translation of mRNAs [59]. Because of the essential roles of MiR-214 in coordinating tumor proliferation, stemness, angiogenesis, invasiveness, extravasation, metastasis, resistance to chemotherapy, and microenvironment, MiR-214 becomes a molecular hub involved in the control of cancer networks. MiR-214 switching in diverse forms of cancer either by its upregulation or downregulation sheds light on the mechanism of its tumorigenic and suppressive roles and has resulted in it being a potential diagnostic/prognostic biomarker and target for therapeutic intervention [48].

MiR-199a is also transcribed as antisense of dynamin 3 (chromosome 1q24.3). Hypermethylation of this region was deemed to correlate with miR-199a in testicular cancer cells and leaded to suppression of cell growth, cancer migration, invasion and metastasis [60]. Like MiR-214, MiR-199a is also either up-regulated or down-regulated in a variety of cancers. For example, it is downregulated in human HCC, liver cancer cells [61] and in testicular germ cell tumor (TGCT) [62]. Interestingly, MiR-199a and miR-214 were concordantly expressed in a human neuron-committed teratocarcinoma NT2 cell line and TGCT patient tissue samples [62]. MiR-199a and MiR-199b (MiR-199a/b) family of miRNAs are conserved within their intronic sequences [63]. The miR-199a/b family is composed of three members, MiR-199a1, MiR-199a2 and MiR-199b, which are transcribed from conserved antisense intronic transcripts of the DNM2 locus (human chromosome 19), DNM3 locus (human chromosome 1) and DNM1 locus (human chromosome 9), respectively. MiR-199a and b regulate endocytic transport by controlling the expression of important mediators of endocytosis such as clathrin heavy chain, Rab5A, low-density lipoprotein receptor (LDLR), and caveolin-1 (Cav-1) [63]. One of the two mature microRNA species derived from MiR-199a, named MiR-199a-5p is associated with tumor malignancy. Both MiR-199a and b-5p, inhibit clathrin-mediated endocytosis through the regulation of clathrin heavy chain, Rab5A and Rab21 expression, affecting the normal function of receptors located in the plasma membrane such as LDLR and transferrin receptor. In summary, MiR-199a/b-5p regulates related physiological processes to those controlled by the host genes in which they are encoded and a significant number of predicted target genes for MiR-199a/b-5p were associated with cellular transport [63]. Moreover, MiR-199a/MiR-214 forms miR-199a/miR-214/PSMD10/TP53/DNMT1 self-regulatory network [62], which might be a potential therapeutic target in the treatment of TGCT [62]. Interestingly, MiR-199a encoded from the opposite strand of dynamin 2 gene DNM2 exerts reciprocal negative regulation upon HIF-1α and HIF-2α [64]. Overexpression of MiR-199a decreased HIF-1α and HIF-2α, cell migration, and metastasis, thus, serves as a regulatory loop between endocytic pathway and hypoxic response in tumor cells. Mir-199a has also been implicated to modulate intracellular iron levels by targeting HIF and indirectly affecting the tumor microenvironment through suppression of lysyl oxidase protein expression in ovarian cancer cells [64].

Apart from MiR-214, MiR-3120 is also encoded from a single gene locus of the dynamin 3 gene, under certain conditions, due to imperfect base pairing. MiR-214 and MiR-3120 are the two distinct microRNAs produced from the fully complementary DNA strands. MiR-3120 is produced following transcription and mRNA processing, whereas MiR-214 is produced by antisense transcription. MiR-3120 regulates heat shock cognate protein 70, thus it serves as a mirror microRNA regulating endocytic function of Hsc70 and auxilin as well as their expression to influence vesicle uncoating. MiR-3120 is also known to prevent uncoating of clathrin-coated vesicles, and MiR-214 is known to target PTEN (phosphatase and tensinhomolog), which interacts with dynamin and regulates synaptic proteins involved in receptor cycling and synaptic plasticity [65]. Despite MiR-3120 being regulator for endocytosis pathways, it is not known whether there is any direct modulation role in the tumorigenosis.

## THERAPEUTIC POTENTIAL OF DYNAMIN-RELATED PROTEINS

Dynamin-related proteins are a group of GTP-binding proteins containing good conservation of the five domains architectures defined for classical dynamin. The five domains contained by classical dynamin are the followings: GTPase domain; middle domain; pleckstrin-homology (PH) domain; effector domain (GED); proline-rich domain (PRD) (Figure 2B). Dynamin-related protein proteins also function in vesicle budding, trafficking and membrane-scission events. These proteins have similarity in some domains but might miss out several and have additional domain conferring distinct roles.

### Dynamin-related protein 1 (Drp1) as a therapeutic target

Drp1, an 80 kDa GTPase without the PRD (Figure 2B), is involved in mitochondrial fission and anti-cancer drug-mediated cytotoxicity, implicating an association with cancer progression [25]. Drp1 is also involved in conferring chemotherapy resistance, cell cycle progression, genome instability, cell migration and apoptosis. It is upregulated in certain types of cancerssuch as lung and breast cancer [25, 26, 66]. Drp1 upregulation and activation induce changes in mitochondrial dynamics crucial to confer the drug resistant in T-cell acute lymphoblastic leukemia cells [67]. The influence of mitochondria on human health and disease and mitochondrial dysfunction in cancer has expanded to include defects in mitochondrial genomics and biogenesis, apoptotic signalling and mitochondrial dynamics [68]. Moreover, defects in mitochondrial fission protein Drp1 are linked to apoptotic resistance and autophagy in a lung cancer model [69]. It has been implicated that drugs targeting to Drp1-mediated mitochondrial fission prevents tumor cell cycle progression. Thus, Drp1 inhibition, or Drp1 knockdown resulted in an observed reduction of cancer cell proliferation and an increase of spontaneous apoptosis and thus serves as an effective therapy for cancer treatment [26]. Drp1 is also found to contribute to the migration of human glioblastoma cells under hypoxia, and causes the poor prognosis of glioblastoma due to its mitochondrial control in brain tumor initiating cells [70]. Nuclear Drp1 may increase drug resistance during hypoxia. Drp1 co-precipitated protein, the human homologue of the yeast repair protein RAD23 (hHR23A), is essential for nuclear transportation of Drp1 [71]. Meanwhile, the nuclear and nucleolar Drp1 (Drp1nuc) are associated with poor cancer prognosis [71]. In brain cancer, Drp1 controls the migration and neuronal differentiation of subventricular zone-derived neural progenitor cells [72]. Inhibiting Drp1 activity induces alteration of the typical migratory cell morphology into round shapes while the polarized mitochondrial distribution was maintained. With these changes, adult neural stem cells (aNSCs) derived from the subventricular zone of the brain failed to migrate, and neuronal differentiation was prevented [73]. Targeting Drp1 activity by genetic approach or pharmacologic inhibitors attenuates growth of stem-like tumors and lead to good prognosis [74], thus, Drp1 serves as a potential target for cancer treatment.

### Mitofusin (Mfn) as a therapeutic target

Mfn 1 and Mfn 2 are involved in fusion between mitochondria by tethering adjacent mitochondria (Figure 2B). These proteins have two transmembrane segments that anchor to the mitochondrial outer membrane. Mfn1 and Mfn2 exist as both homotypic and heterotypic oligomers, and therefore can cooperate as well as act individually to promote mitochondrial fusion. Each isoform is essential for embryonic development and mitochondrial fusion, and both have distinct and redundant functions to promote mitochondrial fusion [17]. Mutations in mitofusin proteins result in fragmented mitochondria, but this can be reversed by mutations in mammalian Drp1. Human lung cancer cell lines exhibit an imbalanced expression level of Drp1/Mfn2, which promotes a state of mitochondrial fission [25]. Lung tumor tissue samples from patients demonstrated a similar increase in Drp1 and decrease in Mfn2 when compared to adjacent healthy lung. Furthermore, Drp1 also regulates cell cycle progression, genome instability, cell migration and apoptosis in cancer cells. These findings raise the possibility of targeting Drp1-mediated mitochondrial fission as an effective therapy for treating cancer [26].

### Dynamin-like GTPase Opa1 as a therapeutic target

Another dynamin family members called Opa1, is localized on mitochondrial intermembrane space, where it facilitates fusion between mitochondria [21]. In various forms of cancer, Opa1 is highly expressed and indicates poor prognosis (Table 1). The mitochondria fusion protein Opa1 expression level is critical in determining the sensitivity of HCC to sorafenib-induced apoptosis in HCC treatment [75] and decreased expression of Opa1 is associated with the treatment of HCC by sorafenib, a multi-kinase inhibitor [75].

## DYNAMIN-TARGETED INHIBITORS FOR CANCER TREATMENT

Targeting dynamin function has great potential to produce a safe therapy to hopefully increase the life expectancy and quality of life for cancer patients. Some dynamin inhibitors have already been moved from the lab into clinical trials. The specificity and potency are the major selection criteria for basic and therapeutic proposes.

Dynamin inhibitors represent a new class of targeted anti-mitotic compounds (Table 2). They are a small but expanding ‘palette’ of compounds available to rapidly and reversibly block dynamin via distinct mechanisms of action, by operating at different stages in its cycle of GTPase activity. Overall, they possess both antimitotic and anticancer effects. The first reported dynamin inhibitors were long-chain ammonium salts called MiTMAB™ compounds [76], and then, followed by dimeric tyrphostins [77], and a series of room temperature ionic liquids (RTILs) [78]. Two of the most potent inhibitors from the long-chain ammonium salts, myristyl trimethyl ammonium bromide (MiTMAB) and octadecyltrimethyl ammonium bromide (OcTMAB), are potent and reversible inhibitors of endocytosis in neuronal and non-neuronal cells, and selectively block dynamin's second function in cytokinesis [79]. Targeting cytokinesis with dynamin inhibitors may be a promising new approach for the treatment of cancer [80, 81]. MiTMABs inhibit dynamin GTPase activity by targeting its PH domain, a module common to many proteins. In contrast to the classical (e.g. taxol) and targeted (e.g. aurora kinase and polo-like kinases inhibitors) anti-mitotic compounds which aim to disrupt the mitotic spindle, advantages of MiTMABs is that it is a cell-permeable, water soluble dynamin 1 and 2 inhibitor which exclusively block cytokinesis without disrupting progression through any other stage of mitosis [80]. Apart from inducing cytokinesis failure, MiTMABs also induce apoptosis in cancer cells [82]. Overall, MiTMABs are highly cytotoxic and possess anti-proliferative properties, which seem to be selective for cancer cells.

**Table 2:** Commonly used dynamin inhibitors are not isoform specific for cancer treatment

Inhibitors	Molecular formula	Targets	Function
MiTMAB	C17H38BrN	Dynamins1 and 2	Surface-active, small molecule, dynamin inhibitors that block endocytosis. targets the dynamin-phospholipid interaction to block dynamin recruitment to membranes but not dynamin oligomerization [79]
OcTMAB	C21H46BrN	Dynamins1 and 2	Works together with MiTMAB to induce cytokinesis failure and inhibit cell proliferation [105]
Dynole 34-2	C25H36N4O	Dynamins1 and 2	More efficacious and has less off-target effects than MiTMABs [81]. It acts on the dynamin G domain [106]. It is not GTP competitive [106]. Inhibit the activity of dynamin following recruitment of dynamin to plasma membranes [83]
Dynasore	C18H14N2O4	Dynamins1 and 2	Noncompetitive inhibitor which dose-dependently inhibits the GTPase activity of dynamin 1, 2, and Drp1 (mitochondrial) [4, 107–109]
Dyngo	C18H14N2O5	Dynamins1 and 2	Greatly improved dynasore analogues, with greatly reduced non-specific in vitro binding and improved potency [78]; it is more potent than dynasore and more specific to block dynamin function after its recruitment, with more versatile cell biology tools with reduced cytotoxicity [4, 78]
Mdivi1	C15H10Cl2N2O2S	Drp1	A selective cell-permeable inhibitor of mitochondrial division by blocking dynamin GTPase activity [110–113]
Iminodyn-22	C23H20N4O8	Dynamins1 and 2	Binds to the GTPase domain at an allosteric site and displays uncompetitive antagonism with respect to GTP[114]
RTIL-13	C30H55BrN2O3	Dynamins1 and 2	Inhibits dynamin I and II GTPase and targets pleckstrin homology (PH) (lipid binding) domain [78]

In contrast to MiTMAB, OcTMAB blocks dynamin recruitment to membranes whereas the Dynole™ compounds and dynasore block dynamin after its recruitment [83]. Dynoles cause cytokinesis failure by blocking abscission, which is consistent with inhibition of dynamin 2. Dynole 34-2 is a novel antimitotic compound which specifically acts at the abscission stage and selectively targets dividing cells. It is a dynamin inhibitor and is proved to be the most potent compound for cytokinesis failure [83]. The dynoles are thought to bind an allosteric site in the GTPase domain and are expected to be more efficacious and have less off-target effects than MiTMABs. Inhibition of dynamin by dynole 34-2 induces cell death following cytokinesis failure in a range of cancer cells, such as cellosaurus cell line SMA-560, paediatric solid tumor line SJ-G2 (glioblastoma), colon cancer cell line SW480, breast cancer cell line MCF-7, human ovarian A2780 cancer cells and HeLa cells [81].

Dynasore and its derivatives are also suitable candidates for potent anti-cancer drugs. Dynasore is a non-competitive inhibitor which dose-dependently inhibits the GTPase activity of dynamin 1 and 2, and presumably at higher doses, other dynamin family members. It also interfered with the *in vitro* GTPase activity of Drp1 [84]. For instance, dynamic remodelling of actin filaments is the basis for a variety of cellular events including cell motility, cancer cell invasion, and the regulation of actin dynamics [85]. Furthermore, invasion activity of H1080 cell, a lung cancer cell line, was suppressed by approximately 40% with dynasore treatment [86]. These results strongly suggest that dynasore potently destabilizes F-actin. Notably, dynasore destabilizes F-actin and inhibits serum-induced lamellipodia formation in cells, disrupts co-localization of dynamin 2 and cortactin and inhibits invasion of lung cancer cell line H1080 [86]. Dynago are greatly improved dynasore analogues with greatly reduced or no non-specific *in vitro* binding and improved potency, they are more potent than dynasore and more specific, blocking dynamin function after its recruitment, with more versatile cell biology tools with reduced cytotoxicity. Among them, Dyngo-4a is the most potent one with 37 fold more potent than dynasore [78].

Two Drp1 inhibitors, mitochondrial division inhibitor 1 (mdivi1) or MiR-499, are potent in inhibition of mitochondrial division [87]. Mdivi1 is a selective cell-permeable inhibitor of mitochondrial division Drp1 and dynamin 1. It enhances death receptor-mediated apoptosis in human ovarian cancer cells and is able to increase the sensitivity of human ovarian cancer cells to death receptor ligands including TRAIL, FAS ligands, and TNF-α [88]. It has been reported to enhance the apoptotic effect of the death receptor ligand TRAIL in human ovarian cancer [57]. Low serum MiR-499 expression was associated with advanced tumor node metastasis (TNM) stage and poor prognosis [89]. Overexpression of MiR-499-5p inhibited cell proliferation and induced apoptosis *in vitro* as well as *in vivo.* Thus, it functions as a tumor suppressor [90]. Other drugs such as micheliolide may also influence Drp1 function. Micheliolide is a guaianolide sesquiterpene lactone, which induced Drp1-mediated cell death in breast cancer cells (MCF-7) through the reactive oxygen species mediated mitochondrial apoptotic pathway [57]. Other dynamin inhibitors include a group of antidepressant drugs which have anti-proliferative activity, for example, sertraline which significantly inhibited tumor growth in CD1 nude mice xenografted subcutaneously with HT29 cells and has also been successfully used in cancer patients [91]. The mechanism involved could possibly be that this group of inhibitors block dynamin activity and inhibit endocytosis in tumor cells, as observed in HeLa and human neuroblastoma SH-Sy5Y cells [92]. Chlorpromazine marketed under the trade names Thorazine and Largactil among others, is a cationic amphiphilic phenothiazine-derived anti-psychotic drug which inhibits endocytosis by blocking dynamin 2 function [93]. Apart from above-noted dynamin inhibitors, chlorpromazine has been shown to have a relative high specificity for brain cancer [94, 95]. Moreover, it has been proven to inhibit glioblastoma growth in rat brains when used in combination with Nitrosureas. Phenothiazine-derived antipsychotic drugs (APDs), which are the cationic amphi-pathic drugs, also inhibit clathrin-mediated endocytosis [93]. It is proposed that potent dynamin inhibition is a shared characteristic of phenothiazine-derived APDs, but not other typical or atypical APDs [93].

Successful cancer treatment requires multiple approaches to reach the most effectiveness. For example, no single combination regimen has clearly emerged as a favourite for the treatment of recurrent or progressive glioblastoma. For instance, the newly diagnosed glioblastoma is now commonly treated with surgery, if feasible, or biopsy, followed by radiation plus concomitant and adjuvant temozolomide, the drug serves as the first line treatment for glioblastoma. However, only approximately one-half of patients respond to temozolomide as it showed little effect in elderly patients with advanced age. Nitrosoureas only remain a second-line treatment option in single and combination regimens, and temozolomide in combination with cisplatin, fotemustine, interferon, sorafenib, celecoxib, irinotecan, or procarbazine/lomustine/vincristine has not been demonstrated to be more effective than temozolomide alone [96]. Similarly, HER1/EGFR tyrosine kinase inhibitors such as erlotinib or gefitinib are the most clinically advanced HER1/EGFR-targeted agents for the treatment of glioblastoma. However, both inhibitors only treat a fraction of patients because HER1/EGFR are only overexpressed in these patients [97]. The newly developed inhibitors for dynamin and its related proteins might offer an alternative option for combination of therapeutics to achieve the synergistic effectiveness for glioblastoma.

## CONCLUSIONS AND PERSEPCTIVES

To conclude, dysregulation of endocytosis has been accepted as an emerging feature of cancer development. Significant evidence points to dynamins and their-related proteins as having an important function in carcinogenesis. Each dynamin isoform contributes to various types of cancer development by regulating endocytosis, cytokinesis, cell migration, invasion, and prognosis. In general, dynamin 1 and 2 are overexpressed in cancerous tissue and each is related to specific cancer types. Targeted therapeutics are required to decrease their expression levels or prevent the hypermethylation of the dynamin 3 gene or inhibitors selectively block the signalling cascades would be beneficial. Dynamin 3 serves as a tumor suppressor in hepatocellular carcinoma pathogenesisis and its expression is decreased during the disease condition due to gene hypermethylation. Targeted therapeutics are required to improve its expression level or blockade the hypermethylation of the DNM3. Moreover, the expression levels of dynamin gene-related proteins become imbalanced in cancer. These proteins are potential diagnostic/prognostic biomarkers and potential targets for therapeutic intervention. Anti-sense non-coding RNAs for the dynamin gene are bifunctional. Up- or down-regulation of non-coding RNA controls dynamin gene expression and mitochondrial division, to promote tumorigeneses or suppress tumorigenesis. Although numerous dynamin inhibitors have been developed so far, they have failed to selectively block a specific isoform of dynamin. Current dynamin inhibitors are non-selective to a particular isoform, thus, selective inhibitors against distinct isoform are needed for future anti-cancer drug development. The most commonly used dynamin inhibitors have revealed off-target effects in dynamin triple knockout cells [98], warranting future development of isoform-specific inhibitors for dynamin as potential therapeutics for successful anti-cancer treatment.

## Abbreviations

aNSCs: adult neural stem cells
AP2: clathrin adaptor protein 2
APDs: Phenothiazine-derived antipsychotic drugs
BoNTs: botulinum neurotoxins
Cav-1: caveolin-1
CCPs: clathrin-coated pits
CME: clathrin-mediated endocytosis
CLTC: clathrin heavy chain
DNM1: DNM2 and DNM3. dynamin 1, 2, 3 genes
Drp1: dynamin-related protein 1
EGFR: epidermal growth factor receptor
Fis1: mitochondrial fission 1
GED: GTPase Effector Domain
HCC: hepatocellular carcinoma
LDLR: low-density lipoprotein receptor
mdivi1: mitochondrial division inhibitor 1
MIS: mitochondrial import sequence
MiTMAB: myristyl trimethyl ammonium bromide
MSP-PCR: methylation specific polymerase chain reaction
OcTMAB: octadecyltrimethyl ammonium bromide
OPA1: dynamin-like GTPase protein Optic Atrophy 1
PCA: Progressive prostate cancer
PH domain: pleckstrin homology domain
PRD: proline rich domain
shRNAs: short hairpin RNAs
TRAIL: TNF-related apoptosis-inducing ligand.
